# DEVELOPMENT AND APPLICATION THE QUESTIONNAIRE FOR MEDICAL CHECKUP OF OLD-OLD (QMCOO) - TAIWAN VERSION

**DOI:** 10.1093/geroni/igac059.2711

**Published:** 2022-12-20

**Authors:** Jui-Ying Hung

**Affiliations:** Chaoyang University of Technology, The National Center for Geriatrics and Gerontology, Wufeng, Taichung, Taiwan (Republic of China)

## Abstract

In 2022, population over the age of 65 is currently about 3.8 million (accounting for about 16% of the total population) as an “aged society” in Taiwan. According to the report about the frailty of the elderly increases with age, especially 75 - 79 years old, are the most obvious functional decline (The Ministry of Health and Welfare, 2022). This trend that also happened in 2005, the interim report of the 3rd national health promotion measures showed an increased prevalence of diabetes mellitus and obesity in Japan. In response to this, Dr. Satake and Dr. Arai (2020) develop the questionnaire for Medical Checkup of Old-Old (QMCOO) suited measure frailty to people aged ≧75 years better and composed of 15 questions regarding the following 10 domains: health condition, mental health, eating behavior, oral function, body weight loss, physical function and falls, cognition function, smoking, social participation and social support implemented in April 2020. This study authorized by Dr. Satake et, al in August 2022, aims to translate the QMCOO in Chinese version and through expert discussions to confirm that there are no difference between the original version in Japanese/English, and the apply query specification for QMCOO in Taiwan. This study recruited 300 older adults in Taichung city to answer all the questions in QMCOO and analysis the diagnostic frailty distributed status compared to the data measure in the Kihon Checklist in the same time. This finding the cutoff scores 2/3 for the QMCOO might be effective in diagnosing frailty in Taiwan.

